# Prevalence of Laryngomalacia among Young Children Presenting with Stridor in a Tertiary Care Hospital

**DOI:** 10.31729/jnma.5244

**Published:** 2020-10-31

**Authors:** Apar Pokharel

**Affiliations:** 1 Department of Ear, Nose, and Throat, and Head and Neck Surgery, College of Medical Sciences, Bharatpur, Nepal

**Keywords:** *laryngomalacia*, *Nepal*, *stridor*

## Abstract

**Introduction:**

Laryngomalacia is one of the most common causes of stridor in young children. It can be a serious concern to both parents and caregivers. The main objective of this study is to find the prevalence of laryngomalacia among young children presenting with stridor in a tertiary care hospital in central Nepal.

**Methods:**

A descriptive cross-sectional study was carried out form 1st December 2017 to 1st May 2020 in children less than two years of age in a tertiary care hospital. Ethical approval was taken from the Institutional Review Committee of the hospital (reference number: 2020/23). Convenient sampling was done. Detailed demography, clinical examination, and video laryngoscopy findings were evaluated to find the prevalence of laryngomalacia among all children with stridor. Data were analyzed by using Statistical Package for the Social Sciences version 20. Point estimate at 95% confidence interval was calculated along with frequency and proportion for binary data.

**Results:**

Out of 430 participants who presented with stridor, the laryngomalacia was found in 234 (66%) (58.7-74.07) cases at a 95% confidence interval. The male: female ratio was 1.7:1. Most children, 192 (67.6%), presented with a milder form of laryngomalacia. The most common type was a mixed type of laryngomalaciain 159 (56%). Sleep-disordered breathing was seen in 113 (39.79%) of children diagnosed with laryngomalacia.

**Conclusions:**

Our study concluded that laryngomalacia was the most common cause of stridor in children less than two years of age. However, in most cases, the problem is not serious and a regular follow-up with weight monitoring is warranted.

## INTRODUCTION

Laryngomalacia is the cause of 45-75% of all congenital stridors.^1^ The stridor is a high-pitched sound created by airflow through the partially obstructed airway. In laryngomalacia, the supraglottic structures are weak and they collapse into the airway during the inspiration resulting in stridor. Mostly the stridor in laryngomalaciais mild which resolves by itself by 12-24 months.^1^ However, the condition must be diagnosed and must be differentiated from other causes of stridor.

No previous study has been done to find the prevalence of laryngomalacia in Nepal. Laryngomalacia is a benign condition of stridor and in most of the cases, no active intervention is needed.^1^ So this condition should always be kept in mind when young children present with stridor so that unnecessary interventions are avoided.

Thus, the study aims to find out the prevalence of laryngomalacia among young children presenting with stridor in a tertiary care hospital in central Nepal.

## METHODS

This was a descriptive cross-sectional study conducted in the Department of Ear, Nose, and Throat, and Head and Neck Surgery (ENT), College of Medical Sciences from 1st December 2017 to 1st May 2020 in the children aged less than two years. Ethical approval was taken from the Institutional Review Committee (reference number: 2020/23). Children aged less than two years who presented to the outpatient or emergency department with stridor were included in the study. The children greater than two years presenting with or without stridor were excluded from the study. Convenient sampling was done. The source of the initial identification of the patient was the hospital record database. The sample size was calculated using the formula,

$$\begin{array}{ll} \text{n} & \text{= }{\text{Z}}^{\text{2}}\times \text{p}\times \text{q}/{\text{e}}^{\text{2}}\\ & \text{= }{\left(1.96\right)}^{\text{2}}\times (0.5)\times (1-0.5)/{\left(0.05\right)}^{\text{2}}\\ & \text{= }384.16\\ & =384 \end{array}$$

where,

n = sample size
p = prevalence of laryngomalacia taken as 50%
q = 1-p
e = margin of error, 5%
Z = 1.96 at 95 % Confidence Interval

Taking a non-response rate of 10%, the sample size was 423. However, the total sample size taken was 430.

After explaining the purpose, importance, and procedure in detail a written informed consent was obtained from all the participants. The protocol approved by the Ethics and Research Committee of the institution was followed according to which children with a diagnosis of respiratory stridor were submitted to detailed anamnesis, flexible video nasolaryngoscopy, and if necessary, rigid bronchoscopy. In the anamnesis, parents were asked about the onset of stridor, aggravating and relieving factors, perinatal history, birth weight, presence of other congenital anomalies, breastfeeding difficulties, weight gain or loss, cyanosis, apnea, and episodes of recurrent upper respiratory tract infections that required hospitalization. This was followed by a detailed examination of the child which included baby weight, chest, and cardiovascular examination, and ear nose, and throat examination. Flexible nasopharyngolaryngoscopy using a 3.2mm diameter flexible fiber in the outpatient setting was then done on these children. Flexible nasopharyngo laryngoscopy was not introduced beyond the laryngeal inlet. However, if abnormal tracheal or bronchial pathology was suspected, then the procedure was performed in the operating room under sedation and spontaneous ventilation. After the diagnosis of laryngomalacia was made, it was again classified as per Onley, et al.(1, 2, 3, or mixed). Type 1 was the posterior collapse of redundant arytenoid mucosa over the arytenoids and cuneiform cartilage. The type 2 laryngomalacia was a lateral collapse of supraglottis secondary to shortened aryepiglottic folds. The type 3 laryngomalacia was the anterior collapse of supraglottis during inspiration due to retroflexed epiglottis.^2^ The type of laryngomalacia was determined through clinical chart recording and video recording.

The laryngomalacia was also classified based upon severity. The mild form had inspiratory stridor with no feeding problems. The resting oxygen saturation was from 98% to 100%. The moderate form was characterized by frequent feeding difficulties. Their oxygen saturation was also lower than the mild form. The child used accessory muscles of respiration for breathing. The severe form of laryngomalacia presented with apneic spells with oxygen saturation going down upto 85%. The children would have feeding difficulties, failure to thrive, and a history of recurrent pneumonia.^3^

Selection and information bias was minimized as much as possible by collecting data in the appropriately predesigned proforma, point estimated at 95% of Confidence Interval calculated along with the frequency for binary data. Data were analyzed using Statistical Package for the Social Sciences (SPSS)version 20.

## RESULTS

A total of 430 children with stridor were recruited in the study. Based on history, clinical examination, and nasopharyngolaryngoscopy, 284 (66%) (58.7-74.07) children (at a 95% confidence interval) were found to be having laryngomalacia (Table 1).

**Table 1:** Different causes of stridor among young children (n = 430).

Variables	n (%)
Congenital	
Laryngomalacia^*^	284 (66%)
Vocal cord palsy	1 (<1%)
Subglottic stenosis	1 (<1%)
Laryngeal web	1 (<1%)
Laryngeal clefts	1 (<1%)
Tracheomalacia	3 (<1%)
Acquired	
Croup	91 (21%)
Epiglotittis	23 (5%)
Subglottic foreign body	4 (<1%)
Retropharyngeal abscess	21 (5%)
Subglottic stenosis	3 (<1%)

* 3 patients of laryngomalacia had synchronous airway lesions. All three cases were of tracheomalacia

Among 284 (66%) children with laryngomalacia, 179 (63.02%) children were male (male: female = 1.7). About 188 (66.19%) children were of Aryans race. About 149 (52.46%) children had a birth weight between 2.5-4kg, which was followed by less than 2.5 kg group which consisted of 114 (40.14%) children (Table 2).

**Table 2:** Demography and clinical features of laryngomalacia in children (n = 284).

Variables	n (%)
Time of onset of stridor	
Since birth or within a week	74 (26.05%)
One week to three months	205 (72.18%)
After 3 months	5 (1.76%)
Ethnicity	
Aryans	188 (66.19%)
Mongoloids	96 (33.80%)
Gender	
Male	179 (63.02%)
Female	105 (36.97%)
Birth weight	
Less than 2.5 kg	114 (40.14%)
2.5-4 kg	149 (52.46%)
More than 4 kg	21 (7.39%)
Cried immediately after birth	222 (78.16%)
Mean Apgar score at 5 mins	3/5
Delivery	
Term	167 (58.80%)
Preterm	112 (39.44%)
Postterm	5 (1.76%)
Method of delivery	
Vaginal delivery	112 (39.44%)
Emergency Caesarian section	119 (41.90%)
Elective Caesarian section	53 (18.66%)
History of neonatal morbidity(n = 69)	
Recurrent pneumonia	27 (9.50%)
Sepsis	22 (7.7%)
Meningitis	5 (1.76%)
Neurological conditions	15 (5.28%)
Feeding problems	36 (12.6%)
History of sleep-disordered breathing	113 (39.79%)
Type of laryngomalacia	
Type 1	76 (39.79%)
Type 2	37 (13.03%)
Type 3	12 (4.23%)
Mixed	159 (55.99%)
Severity of laryngomalacia	
Mild	192 (67.6%)
Moderate	87 (30.63%)
Severe	5 (1.76%)
Synchronous airway lesions	3 (1.06%)

Almost 216 (76%) of children with laryngomalacia didn't show sufficient weight gain during the time of presentation in the ENT outpatient department. The most common time for the onset of stridor for laryngomalacia was from 1 week to 3 months of birth. About 222 (78.16%) children had a history of crying immediately after birth. Almost 167 (58.80%) babies with laryngomalacia were term deliveries and the most common method of delivery was an emergency Caesarian section seen in 119 (41.9%). Nearly 69 (24.29%) children with laryngomalacia had a history of neonatal morbidity with episodes of recurrent pneumonia being the most common. About 36 (12.6%) children presented with feeding problems like coughing, choking, regurgitation with feeding, and slow oral intake.

Sleep-disordered breathing was seen in 113 (39.79%) of children diagnosed with laryngomalacia. A mixed type of laryngomalacia was seen in most of the children 159 (56%) followed by 76 (39.79%) type 1. About 192 (67.6%) of the children presented with a mild form of laryngomalacia. The indication of surgery was present in only 5 (1.76%) children. However, parents of all five children refused for any operative interventions. Three patients of laryngomalacia had synchronous airway lesions and all three cases were of tracheomalacia.

## DISCUSSION

The incidence of laryngomalacia was seen in 66% of all children less than 2 years of age who presented with stridor. The finding was similar to a study by Richter et al which showed a prevalence of 45-75%.^1^

The demographics of the study population in our work were comparable to those of other series. Our study showed a 1.7:1 male to female ratio. This was consistent with the male predominance reported in other LM series.^4–6^ However, in a study done by Edmondson, et al. the male to female ratio was almost equal.^7^ Around two-thirds of the patients were of the Aryan race. Some studies showed a predominance of laryngomalacia among Caucasian infants, while others showed racial diversity playing no role in the occurrence of laryngomalacia.^7,8^ The most common age of presentation in our study was between 1 week and 3 months. This finding was similar to other case series describing classic laryngomalacia presentations.^5,6^ However, in a case series by Cooper, et al. the mean age of presentation was 14.5 months. This was because the authors had also included children with sleep-disordered breathing as a primary presentation of laryngomalacia.^9^

According to our results, around 52.46% of children had normal birth weight and 40.14% of children had low birth weight. The weight of the children was also measured during the time of presentation in the hospital. Some studies suggest low birth weight be a strong predictor of laryngomalacia.^7^ However in our study, the majority of the children were of normal birth weight. Almost 76% of children with laryngomalacia didn't show sufficient weight gain. This finding was very much similar to a study done by Kusaket al.^10^ The insufficient weight gain might be due to increased effort of breathing, gastro-esophageal or laryngopharyngeal reflux disease, and uncoordinated suck-swallow-breath sequence. These children had higher caloric demands due to increased respiratory effort but and on the other hand, they might have feeding difficulties.

Most of the children with laryngomalacia were term deliveries. In a study done by Kusak, et al. the majority of cases of laryngomalacia were term deliveries, similar to the findings of our study.^10^ In our study, around 41.90% of the children with laryngomalacia were born by emergency caesarian section. This was in contrast to a study by McSwiney, et al. where the majority of the children were normal vaginal delivery.^11^

The presence of concomitant neonatal morbidity was seen in 24.29% of children. The most common problem faced by these children was recurrent pneumonia in 9.5% of the children. In a study done by Kusak, et al. The prevalence of comorbid conditions among children with laryngomalacia was 27%.^10^ In our study, the presence of feeding problems was seen in 12.6% of children with laryngomalacia. The swallowing assessment was based on the history of feeding problems like coughing, choking, regurgitation with feeding, and slow oral intake. No clinical swallowing evaluations by speech pathologists, modified barium swallow studies, and fiberoptic endoscopic evaluations of swallowing were done which was one of the limitations of this study. In a study done by Simons, et al. The prevalence of swallowing problems among children with laryngomalacia was seen in 50.3% of children. This study also didn't find any significant correlation between the severity of laryngomalacia and medical comorbidities with swallowing dysfunction.^12^ In our study, the problem of sleep-disordered breathing was seen in 39.79% of children. In the study of Lepe, et al. showed 88% of children with laryngomalacia exhibit features of sleep-disordered breathing. The authors even advocated sleep study in every child with laryngomalacia to establish the severity of the problem and the need for non-invasive ventilator support.^13^ Besides, sleep-disordered breathing could be one of the early manifestations of late-onset laryngomalacia in children. The prevalence of laryngomalacia within children presenting with sleep-disordered breathing was 3.9%.^4^

In our study mixed type of laryngomalacia was the most common presentation seen in 56% of children. This was followed by type 1 laryngomalacia 39.79%. Some studies suggest type 1 laryngomalacia (53%) as the most common laryngomalacia followed by mixed type (35%). In our study, the majority of the laryngomalacia cases were of the mild variant (67.6%). Thompson, et al. stated an equal prevalence of both mild and moderate forms of laryngomalacia.^3^ Only 3 cases of synchronous airway lesions were seen in our study and all three cases were of tracheomalacia. Studies show the rate of synchronous airway lesions in laryngomalacia varies from 7.5 to 64% and this rate strongly depends upon the study setting as the tertiary care center would get many such referrals. The most common lesion wastracheomalacia followed by subglottic stenosis and vocal cord palsy.^14-17^

There were certain limitations to our study. First, this was a cross-sectional study. So the evaluation of children with laryngomalacia on follow-up was beyond the scope of this study. As the study was done in a tertiary care center, the population of children with laryngomalacia was highly selected and might not be representative of the general population of children with laryngomalacia.

## CONCLUSIONS

Laryngomalacia is one of the most common differential diagnoses of stridor in young children. Our study showed a similar prevalence compared to other studies done in the past. All infants with stridor require a proper history taking, clinical examination, and visualization of the airway to establish the diagnosis and plan the management. In most of the cases, the children have a mild form of laryngomalacia which warrant a regular follow-up with weight monitoring.

## Conflict of Interest

**None.**
